# The Retention Rate and Safety of Secukinumab as a First-Line Biologic Agent in Axial Spondyloarthritis Compared to a First Tumor Necrosis Factor (TNF) Inhibitor: A Real-World, Longitudinal Study

**DOI:** 10.7759/cureus.70365

**Published:** 2024-09-28

**Authors:** Salma Zemrani, Bouchra Amine, Imane El binoune, Samira Rostom, Latifa Tahiri, Fadoua Allali, Rachid Bahiri

**Affiliations:** 1 Department of Rheumatology A, El Ayachi Hospital, Ibn Sina University Hospital, Salé, MAR; 2 Department of Rheumatology B, El Ayachi Hospital, Ibn Sina University Hospital, Salé, MAR

**Keywords:** effectiveness, retention rate, secukinumab, spondyloarthritis, tnf-inhibitors

## Abstract

Background and objective

Secukinumab (SECU) is a biologic disease-modifying antirheumatic drug (bDMARD) that has demonstrated effectiveness against axial spondyloarthritis (ax-SpA). However, in clinical practice, secukinumab is most commonly used as a second-line treatment after failure of or intolerance to tumor necrosis factor inhibitors (TNFi). In this study, we aimed to compare the two-year drug retention between secukinumab and TNFi in biologic-naïve patients with ax-SpA, to estimate the remission/low disease activity (LDA) rates in both groups and assess the safety profiles.

Methods

This was a longitudinal observational study involving patients with ax-SpA who were biologic-naïve and were receiving SECU or TNFi between December 2019 and December 2021. The two-year therapeutic retention rate in both groups was determined. Remission and LDA rates obtained at 24 months according to the Ankylosing Spondylitis Disease Activity Score based on C-reactive protein (ASDAS-CRP) scale, as well as the safety profile, were compared between the two groups.

Results

Seventy-five patients were included in the study. Of them, 34.6% received SECU, while 65.3% received TNFi; 85.3% were males. The mean age was 37.8 ±9 years, the mean disease duration was 10.2 ±6.1 years, and the initial ASDAS-CRP was 3.5 ±0.8. At 24 months; the therapeutic retention rate was 70% for SECU and 66% for TNFi. The reasons for discontinuation were inefficacy (SECU: 11.5%, TNFi: 20.4%, p=0.33), side effects (SECU: 0, TNFi: 4.1%, p=0.29), and socioeconomic conditions (SECU: 15.5%, TNFi: 10.2%, p=0.51). The rate of patients achieving remission and LDA was comparable between the two groups: (remission - SECU: 23.1%, TNFi: 24.5%, p=0.92; LDA - SECU: 73.1%, TNFi: 73.5%, p=0.16). There was no statistically significant difference in the safety profile.

Conclusions

Our findings suggest that the effectiveness and safety of secukinumab for ax-SpA in biologic-naïve patients are comparable to those of TNFi.

## Introduction

Axial spondyloarthritis (ax-SpA) is a chronic inflammatory rheumatic disease (IRD) with a diverse presentation. It mainly affects the axial skeleton [1], with the potential association with peripheral involvement (arthritis, enthesitis, dactylitis), as well as extra-musculoskeletal manifestations (anterior uveitis, psoriasis, inflammatory bowel disease) [2]. According to international guidelines, the therapeutic objective for ax-SpA is remission or, at least, low disease activity (LDA). To achieve this goal, a tailored treat-to-target (T2T) approach is necessary for each patient [3]. Tumor necrosis factor inhibitors (TNFi) are the preferred biologic treatment for patients with active ax-SpA who have had an inadequate response or are intolerant to non-steroid anti-inflammatory drugs (NSAIDs). However, primary or secondary failure may occur in almost 40% of patients [4].

In recent years, the therapeutic options of ax-SpA have significantly expanded with the advent of new drugs that target the interleukin (IL)‐23/Th17 pathway, which is involved in the disease's pathophysiology [5]. Secukinumab (SECU) is a fully human monoclonal antibody that targets IL-17A [6]. Recently, data from randomized controlled trials (RCTs) (MEASURE and PREVENT) have shown that SECU is effective and well-tolerated compared to placebo in both radiographic and non-radiographic ax-SpA in biologic-naïve patients as well as those who have experienced an inadequate response or intolerance to TNFi [7,8]. However, there is scarce real-life data on its effectiveness as a first-line treatment in ax-SpA. Therefore, TNFi remains the first biologic choice in current practice based on over a decade of use.

The primary objective of our study is to evaluate the therapeutic retention rate of secukinumab in comparison to TNFi in biologic-naïve patients with ax-SpA. The secondary objectives are to assess remission and LDA rates in both groups during the 24-month follow-up period, as well as the safety profile.

## Materials and methods

Study design and population

This was a longitudinal, observational, real-life study conducted at El Ayachi Hospital in Salé, Morocco. The study included all patients aged 18 years or over with ax-SpA who received secukinumab (SECU group) or TNFi (TNFi group) as a first-line biologic agent between December 2019 and December 2021, following the failure of or intolerance to NSAIDs. Patients who had received multiple biological treatments were excluded from the study.

Data collection

Demographic features, comorbidities, disease duration, and disease characteristics including phenotypic presentation, radiographic involvement, and extra-articular manifestations were collected for each patient. History of treatment, particularly the use of conventional disease-modifying anti-rhematic drugs (CsDMARDs) and steroids, was also recorded.

Follow-up

The follow-up period commenced on the date of the initiation of biologic disease-modifying antirheumatic drug (bDMARD) treatment and concluded either on the date of cessation of treatment or at the end of the study.

Treatment retention rate

Retention rates were calculated as the percentage of patients still on TNFi or SECU at 12 and 24 months after treatment initiation.

Treatment response

Effectiveness was assessed at 12 and 24 months of follow-up. Clinical response was evaluated using the Ankylosing Spondylitis Disease Activity Score based on C-reactive protein (ASDAS-CRP) scale. Treatment response was defined as achievement of ASDAS inactive disease (<1.3) or at least LDA (ASDAS-CRP <2.1).

Safety

Safety was assessed in terms of the occurrence of side effects from the time of inclusion until the 24-month visit. A treatment-adverse event was defined as any newly observed event that had not been present before treatment exposure.

Statistical analysis

Statistical analysis was performed using SPSS Statistics, version 13.0 (IBM Corp., Armonk, NY). Normally distributed parameters were presented as mean ±standard deviation (SD), and asymmetric parameters were expressed as median ±interquartile range (IQR, defined as 25-75th percentiles). Qualitative data were presented as frequencies (number and percentage). Retention rate was described by Kaplan-Meier curves during 24 months of follow-up. The comparison between the TNFi group and the SECU group was done using the T-test and Mann-Whitney test for quantitative variables and the Chi-squared test or Fischer’s exact test for qualitative variables. P-values less than 0.05 were considered statistically significant.

Compliance with ethical standards

The study was approved by the Ethics Committee for Biomedical Research Mohammed V University - Rabat (approval number: 37/24). Patients’ information was kept anonymous and written informed consent was received from each patient.

## Results

Baseline characteristics

Seventy-five biologic-naïve ax-SpA patients were included in this study: 26 in the SECU group, and 49 in the TNFi group. TNFi used were infliximab (42,9%), golimumab (16,3%), adalimumab (14,3%), certolizumab (14,3%), and etanercept (12,2%).

The baseline characteristics of the cohort are summarized in Table 1. Of note, 85.3% of the patients were male. The mean age at diagnosis was 38 ±9 years, the mean disease duration was 10.22 ±6.1 years, and the mean BMI was 23 ±3.9 Kg/m^2^;41.3% had a history of smoking, peripheral involvement was found in 34.7%, and enthesitis in 16%. Concerning extra-musculoskeletal manifestations, a history of uveitis was found in 6.7%, and inflammatory bowel disease in 2.7%. In terms of disease activity, ASDAS-CRP before biotherapy was 3.5 ±0.8. There was no significant difference between the two groups in terms of demographic data, comorbidities, disease characteristics, and disease activity.

**Table 1:** Baseline characteristics of patients treated with secukinumab or TNFi and comparison between the two groups ^∗^Disease activity before initiating biologic treatment ASDAS-CRP: Ankylosing Spondylitis Disease Activity Score based on C-reactive protein; BASFI: Bath Ankylosing Spondylitis Functional Index; BMI: body mass index; BSADAI: Bath Ankylosing Spondylitis Disease Activity Index; SD: standard deviation; SECU: secukinumab; TNFi: tumor necrosis factor inhibitor

Variables	Total (n=75)	SECU (n=26)	TNFi (n=49)	P-value
Age, years, mean ±SD	37.8 ±9	37.6 ±13.7	37.9 ±5.2	0.89
Sex, n (%)	64 (85.3)	23 (88.5)	41 (83.7)	0.57
BMI, kg/m^2^, mean ±SD	22.9 ±3.9	23.1 ±3.7	22.8 ±4.1	0.78
Hypertension, n (%)	5 (6.7)	2 (2.7)	3 (4)	0.75
Diabetes, n (%)	3 (4)	1 (3.8)	2 (4.1)	0.96
Smoking, n (%)	31 (41.3)	13 (50)	18 (38.3)	0.33
Disease duration, years, mean ±SD	10.2 ±6.1	11.5 ±7.3	9 ±4.7	0.16
Radiographic ax-SpA, n (%)	60 (80)	20 (76.9)	40 (83.3)	0.5
Non-radiographic ax-SpA, n (%)	14 (18.7)	6 (23.1)	9 (16.7)	0.5
Peripheral involvement, n (%)	26 (34.7)	12 (46.2)	14 (28.6)	0.12
Enthesitis, n (%)	12 (16)	6 (23.1)	6 (12.2)	0.22
Psoriasis, n (%)	0	0	0	-
History of uveitis	5 (6.7)	2 (7.7)	3 (6.1)	0.79
Inflammatory bowel disease, n (%)	2 (2.7)	0	2 (4.1)	0.29
HLA-B27 (positive), n (%)	10 (13.3)	3 (11.5)	7 (14.3)	0.58
History of treated tuberculosis, n (%)	7 (9.3)	3 (11.5)	4 (8.2)	0.63
Latent tuberculosis, n (%)	8 (10.7)	4 (15.4)	4 (8.2)	0.33
BASDAI^*^, mean ±SD	4.5 ±1.5	4.2 ±1.4	4.7 ±1.6	0.31
ASDAS-CRP^*^, mean ±SD	3.5 ±0.8	3.4 ±0.8	3.6 ±0.9	0.53
BASFI^*^, mean ±SD	5.2 ±2.3	4.9 ±2.6	5.3 ±2.2	0.59

Retention rate and treatment response

**Figure 1:**
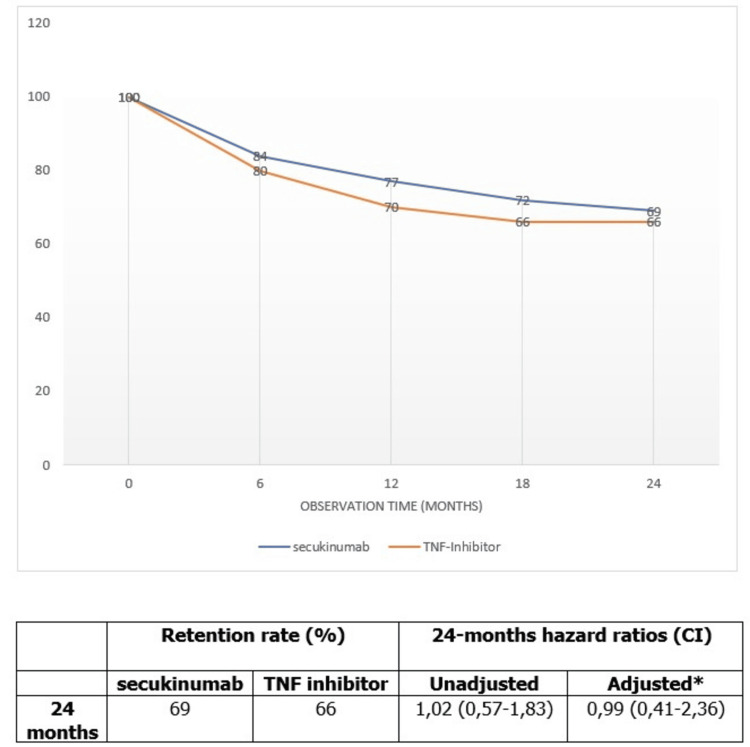
Kaplan–Meier curves for drug retention at 24 months for secukinumab and TNFi *Values adjusted for age, sex, baseline ASDAS-CRP, and fibromyalgia The graph includes adjusted and unadjusted hazard ratios (HRs) for drug survival in secukinumab versus TNFi patients ASDAS-CRP: Ankylosing Spondylitis Disease Activity Score based on C-reactive protein; TNFi: tumor necrosis factor inhibitor

At 12 and 24 months of follow-up, the retention rate in SECU and TNFi groups was 77%/70% and 69%/66% respectively; there was no statistically significant difference between the two groups (Figure 1).

The reasons for discontinuing treatment were similar between the two groups. In the SECU group, ineffectiveness was reported in 15.5% of patients, compared to 20.4% in the TNFi group. Discontinuation of treatment due to adverse events was not reported with secukinumab. However, it was noted in 4% of patients treated with TNFi. Furthermore, 15.5% and 10.2% of patients in the secukinumab and TNFi groups, respectively, discontinued treatment due to loss of follow-up related to socioeconomic factors (Table 2).

**Table 2:** Reasons for the discontinuation of Secukinumab or TNFi SECU: secukinumab; TNFi: tumor necrosis factor inhibitor

Reason	SECU (n=26), n (%)	TNFi (n=49), n (%)	P-value
Primary ineffectiveness	3 (11.5)	6 (12.2)	0.92
Secondary ineffectiveness	0	4 (8.2)	0.13
Adverse event	0	2 (4.1)	0.29
Others	4 (15.5)	5 (10.2)	0.51

**Figure 2:**
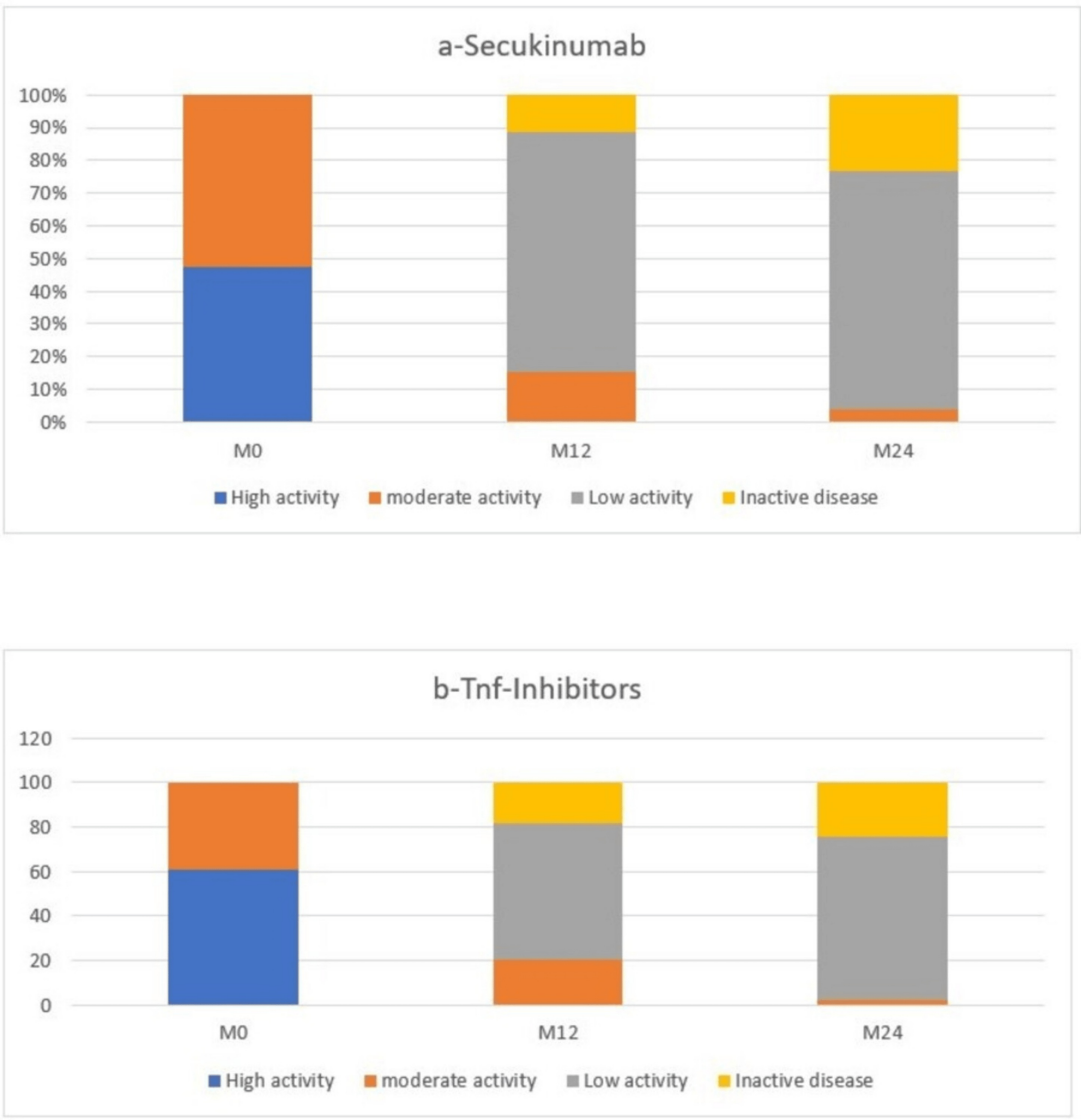
Remission/LDA rates in secukinumab (A) and TNFi (B) groups during 24 months of follow-up LDA: low disease activity; TNFi: tumor necrosis alpha inhibitor

The majority of our patients achieved therapeutic objectives, defined as either remission or LDA according to ASDAS-CRP. The 12-month LDA rate was 73.1% for secukinumab and 61.2% for TNFi (p=0.23), while remission was achieved in 11.5% in the secukinumab group and 18.4% in the TNFi group (p=0.44). Therapeutic response was maintained at 24 months of follow-up. The LDA/remission rates for secukinumab and TNFi were 73.1%/23.1% and 73.5%/24.5%, respectively, with no statistically significant difference (Figure 2 and Table 3).

**Table 3:** Comparison of remission and LDA rates between SECU and TNFi groups LDA: low disease activity; SECU: secukinumab; TNFi: tumor necrosis factor inhibitor

		SECU (n=26), %	TNFi (n=49), %	P-value
12 months	Remission	11.5	18.4	0.44
	LDA	73.1	61.2	0.23
24 months	Remission	23.1	24.5	0.92
	LDA	73.1	73.5	0.16

Safety profile

There was no significant difference between the two groups in the incidence of infusion or injection reactions (p=0.46), coronavirus disease 2019 (COVID-19) disease (p=0.41), active tuberculosis (p=0.46), as well as other respiratory tract infections (p=0.27). Other side effects were noted, particularly two cases of paradoxical uveitis under TNFi. No other serious adverse events were recorded in either group (Table 4).

**Table 4:** Adverse events during follow-up COVID-19: coronavirus disease 2019; SECU: secukinumab; TNFi: tumor necrosis factor inhibitor

Adverse event		Total (n=75), n (%)	SECU (n=26), n (%)	TNFi (n=49), n (%)	P-value
Infections	COVID-19	6 (8)	3 (11.5)	3 (6.12); etanercept (n=1); adalimumab (n=1); infliximab (n=1)	0.41
	Tuberculosis	1 (1.3)	0	1 (2); infliximab (n=1)	0.46
	Other respiratory tract infection	10 (13.3)	4 (15.3)	6 (12.2); etanercept (n=2); golimumab (n=2); adalimumab (n=1); infliximab (n=1)	0.27
	Otorhinolaryngological infections	3 (4)	1 (3.8)	2 (4); certolizumab (n=1); golimumab (n=1)	0.5
	Zona	0	0	0	-
Cancer		0	0	0	-
Cardiovascular events		0	0	0	-
Vasculitis		0	0	0	-
Allergic reaction		1 (1.3)	0	1 (2); infliximab (n=1)	0.46
Paradoxal events		2 (2.6)	0	2 (4); infliximab (n=1); golimumab (n=1)	0.29
Death		0	0	0	-

## Discussion

Effectiveness of secukinumab in bDMARDs-naïve patients with ax-SpA

The effectiveness of secukinumab in patients with ax-SpA was demonstrated in two RCTs (MEASURE and PREVENT). A statistically significant improvement in Assessment of SpondyloArthritis International Society (ASAS)20 and ASAS40 scores compared to placebo was shown in both bDMARD-naïve and refractory patients with radiographic and non-radiographic ax-SpA [7, 8]. Our real-world study demonstrated that the retention rate of secukinumab in biologic-naïve patients with ax-SpA was 69% at 24 months, with LDA and remission rates of 73.1% and 23.1%, respectively.

Currently, there is limited real-world evidence on the effectiveness of IL-17 inhibitors as a first-line bDMARD, with only a few published studies available. In an Indian retrospective study involving 27 patients with ax-SpA with only 19 being biologics-naïve, 65% achieved LDA and 21% achieved remission at 24 months, a finding that aligns with our own [9]. Furthermore, a similar retention rate was found in a retrospective multicentric cohort including 221 patients, in which secukinumab was used as a first-line bDMARD in 38%, with 24 24-month retention rate of 67% [10]. As per another study, based on the data from the EuroSpA registry, 414 out of 1860 ax-SpA patients received secukinumab as a first-line treatment, with a one-year retention rate of approximately 84%, which is higher than the 77% found in our study. Furthermore, 18% and 44% of these patients achieved remission and LDA, respectively [11].

Also, data on secukinumab effectiveness as a first-line bDMARD from RCTs are limited. In a subgroup analysis of the MEASURE 2 study, both bDMARD-naïve and refractory patients showed a statistically significant improvement in ASAS20 and ASAS40 scores compared to placebo [7]. The PREVENT trial was the first RCT to evaluate the efficacy and safety of SECU treatment in patients with non-radiographic ax-SpA. The one-year retention rate was 86.7%, and the ASAS40 response was statistically significant when compared to placebo at weeks 16 and 52 in patients who had not previously received TNFi therapy and in those who had experienced an inadequate response to TNFi therapy [8]. However, we could not compare these findings to our real-world study due to differences in sample size and study design. From another perspective, our finding showed that retention rate and effectiveness during the 24-month follow-up were comparable between secukinumab and TNFi with a hazard ratio (HR) of 0.99 (95% CI: 0.41 to 2.36) after adjusting for age, sex, ASDAS-CRP at baseline, and fibromyalgia.

Studies evaluating the efficacy of secukinumab versus anti-TNF in bDMARDs-naïve patients are scarce. A real-life ax-SpA cohort study has demonstrated that the one-year retention rate was comparable between secukinumab as a first-line bDMARD and adalimumab as a reference (HR: 1.27, 95% CI: 0.99-1.64) [12]. Similarly, data from the DANBIO registry demonstrated that secukinumab and TNFi have similar responses, although secukinumab was used in patients who had previously received TNFi and was used as a first-line treatment only in 1% of cases [13]. As per the EuroSpA registry, the 12-month retention rate in biologic-naïve patients with ax-SpA initiating TNFi was 73% [14], which is similar to the rate of 70% found in our population. These results are comparable to those reported in previous observational studies of patients with ax-SpA who were initiating treatment with a TNFi. In a Swedish study of 112 patients with non-radiographic ax-SpA, the 12-month retention rate was 76%, and the two-year retention rate was 65% [15]. In a study of Danish TNFi-naïve patients with AS, 12-month and 24-month retention rates were 74% and 63% respectively [16].

Reasons for discontinuing treatment

In the study by Sivera et al., discontinuation of secukinumab was related to primary failure in 44%, secondary failure in 30%, adverse events in 10%, and other reasons, including loss of follow-up and patient decision, in 16%. However, this study included both first-line and second-line secukinumab patients [10]. In our cohort, the primary reason for discontinuing secukinumab was ineffectiveness, including both primary and secondary failure, followed by discontinuation due to adverse events. Moreover, there were no statistically significant differences compared to the TNFi group. On the other hand, 15.5% and 10.2% in secukinumab and TNFi groups discontinued treatment due to loss of follow-up related to socioeconomic factors. Hence, it is important to mention that the unavailability of some bDMARDs agents and the lack of global social insurance for all patients are significant issues in our context and may result in non-adherence to treatment.

Safety

Our data did not reveal any serious adverse events during follow-up, and the results were comparable between both groups. Studies evaluating the safety of secukinumab in the real world are rare. Deodhar et al. evaluated the long-term safety by pooling data from clinical trials on ax-SpA, psoriatic arthritis, and psoriasis. The most common adverse events were respiratory tract infections, followed by cutaneous infections; however, there were no reports of systemic infections, and no cases of tuberculosis reactivation were noted. Other side effects were noted in a small proportion of cases, including inflammatory bowel disease, neutropenia, major cardiovascular events, and anti-drug antibodies [17]. Regarding the Indian study mentioned above, only a few adverse events were noted, including upper respiratory tract infection, one case of hepatitis B, and one case of leukocytoclastic vasculitis. However, no case of tuberculosis reactivation was reported [9].

Currently, there is a lack of sufficient comparative safety data available for TNFi and IL-17 inhibitors. The incidence of adverse events between the IL-17 inhibitor and etanercept groups was comparable in the two short RCTs (ACCEPT and FIXTURE) conducted in patients with psoriasis. In light of these findings and according to the available literature data from clinical trials, it can be concluded that IL-17 inhibitors and TNFi are equally safe. Nevertheless, the risk of reactivation of tuberculosis infection remains higher with TNFi [18,19].

To our knowledge, this is the first real-world study to assess the effectiveness and safety of secukinumab in biologic-naïve patients with ax-SpA compared to a first-line TNFi. However, the study has some limitations. Firstly, some data were missing due to the real-world setting of the study. Nevertheless, all efforts were made to gather available data. Secondly, radiographic assessment was not recorded in our population. Additionally, due to the small sample size of patients, we were unable to compare different TNFi classes as well as different secukinumab doses.

## Conclusions

Our study showed that secukinumab and TNFi have a similar retention rate, effectiveness, and safety profile in biologic-naïve patients with ax-SpA. This finding is in line with the latest ASAS/EULAR recommendations for the management of ax-SpA, which position IL-17i as one of the first bDMARD options as per current practice. Nevertheless, larger studies and head-to-head RCTs are needed to validate our findings.
